# Comparison of Dry Versus Wet Milling to Improve Bioethanol or Methane Recovery from Solid Anaerobic Digestate

**DOI:** 10.3390/bioengineering6030080

**Published:** 2019-09-06

**Authors:** Florian Monlau, Cecilia Sambusiti, Abdellatif Barakat

**Affiliations:** 1 APESA, Pôle Valorisation, Cap Ecologia, Avenue Fréderic Joliot Curie, 64230 Lescar, France; 2 UMR, IATE, CIRAD, Montpellier SupAgro, INRA, Université de Montpellier, 34060 Montpellier, France; cecilia.sambusiti@gmail.com (C.S.); abdellatif.barakat@inra.fr (A.B.)

**Keywords:** anaerobic digestion, solid digestate, milling process, sugars recovery, energy balances, bioethanol production

## Abstract

Biogas plants for waste treatment valorization are presently experiencing rapid development, especially in the agricultural sector, where large amounts of digestate are being generated. In this study, we investigated the effect of vibro-ball milling (VBM) for 5 and 30 min at a frequency of 20 s^−1^ on the physicochemical composition and enzymatic hydrolysis (30 U g^−1^ total solids (TS) of cellulase and endo-1,4-xylanase from *Trichoderma longibrachiatum*) of dry and wet solid separated digestates from an agricultural biogas plant. We found that VBM of dry solid digestate improved the physical parameters as both the particle size and the crystallinity index (from 27% to 75%) were reduced. By contrast, VBM of wet solid digestate had a minimal effect on the physicochemical parameters. The best results in terms of cellulose and hemicelluloses hydrolysis were noted for 30 min of VBM of dry solid digestate, with hydrolysis yields of 64% and 85% for hemicelluloses and cellulose, respectively. At the condition of 30 min of VBM, bioethanol and methane production on the dry solid separated digestate was investigated. Bioethanol fermentation by simultaneous saccharification and fermentation resulted in an ethanol yield of 98 g_eth_ kg^−1^ TS (corresponding to 90% of the theoretical value) versus 19 g_eth_ kg^−1^ TS for raw solid digestate. Finally, in terms of methane potential, VBM for 30 min lead to an increase of the methane potential of 31% compared to untreated solid digestate.

## 1. Introduction 

Agricultural wastes represent a vast biomass resource that can readily be converted into sustainable biofuels, chemicals, and other economic by-products by use of a biorefinery concept. As anaerobic digestion has become an efficient technology for waste treatment and renewable energy production, it has resulted in a substantial increase in the use of agricultural anaerobic digesters throughout the EU [1,2]. Anaerobic digestion (AD) is a well-established biological process that has been in use for a long time to anaerobically degrade organic materials into biogas, which is a mixture of CH_4_ (50–70%) and CO_2_ (30–50%), and digestate [3]. After a cleaning process, biogas can be further converted into heat and electricity through cogeneration heat and power (CHP) systems [4]. In parallel, biogas can also be upgraded into biomethane through several technologies (e.g., chemical absorption, membrane separation, water scrubbing, pressure swing adsorption, etc.) as a substitute of natural gas or its use as a transport fuel [3]. Digestate is mainly comprised of water (over 90%), residual undegraded fibers (e.g., cellulose, hemicelluloses, and lignin), and inorganic compounds (e.g., ash), and it is generally mechanically separated into liquid and solid fractions that are stored and handled separately [5,6]. Nevertheless, due to the low hydraulic retention times (HRTs) that are generally applied in industrial biogas plants, after which biogas production starts to decrease, part of the organic matter remains in the solid phase of the digestate [7,8]. 

Consequently, research on alternative valorization routes for solid separated digestate to reduce the environmental impact of disposal and to improve the economic profitability of AD plants is gaining a great interest from the scientific community [5,6]. To date, the valorization strategies that have been investigated are thermochemical processes such as torrefaction and hydrothermal carbonization [9], pyrolysis [4,10], and gasification [11]. Few studies to date have investigated how to use the remaining organic matter present into the solid digestate for sugar platform production and to generate further biofuels such as bioethanol or methane [12,13]. As reported by Santi et al., (2015), digestate produced by commercial agricultural biogas plants still contains a considerable quantity of cell wall polymers [8]. These could potentially be back to AD after a pretreatment step [7] or used in biorefinery processes, for sugar platform production (from C_5_ and C_6_) by enzymatic hydrolysis and further generation of biofuels such as bioethanol [14,15]. 

Nonetheless, due to the recalcitrant properties of the organic matter remaining in solid digestate, a pretreatment step is necessary to improve its biodegradability and further enzymatic hydrolysis and/or biofuel production such as bioethanol or methane by recirculation into the AD process [16,17,18]. Several pretreatment technologies have been tested with agricultural wastes such as physical, thermal, thermochemical, and biological pretreatment, on their own or in combination with each other [17]. Thermal and thermochemical pretreatments have been extensively investigated in terms of their ability to enhance enzymatic hydrolysis and further bioethanol production, although they can also result in potential inhibitors (e.g., furans and polyphenols) generation for microorganisms and strains [19,20]. Another promising option is the use of mechanical pretreatment, which avoids the production of potential inhibitors in addition to improving the physical properties (e.g., the crystallinity, accessible surface area, and particle size) for further enzymatic hydrolysis and/or biofuel production [21,22]. Over the past decades, a number of mechanical size-reduction processes have been developed and investigated including ball mills, vibratory mills, hammer mills, knife mills, colloids mills, two-roll mills, and extruders [21,22].

Although mechanical processes are commonly used in the biorefinery process [21], few studies have investigated the use of mechanical fractionation on solid separated digestate of agricultural biogas plants [12]. Among these fractionation methods, vibro-ball milling (VBM) has the advantages of already being used at the industrial level and of having the capacity to process both dry and wet biomasses. Indeed, when the biogas is valorized through a CHP system, excess heat can be available for drying of the solid digestate [4]. VBM has previously been shown to be a promising technology for alteration of the physical properties (e.g., the particle size and the crystallinity index) and improvement of enzymatic hydrolysis [23,24,25]. Therefore, the aims of this study were the following ones: (i) to evaluate the impact of vibro-ball milling on the physicochemical properties of both dry and wet solid digestates; (ii) to evaluate the effect of vibro-ball milling on the enzymatic hydrolysis performances of dry and wet solid anaerobic digestates; (iii) to derive the energetic balances of the various scenarios investigated; and (iv) to assess the best conditions for bioethanol and methane production compared to raw solid digestate. 

## 2. Materials and Methods

### 2.1. Samples Preparation and Mechanical Pretreatment

Solid separated digestate (SS-DIG) was collected from a mesophilic full-scale AD plant in the Lombardy region of northern Italy. The plant was fed with 95 tons fresh matter per day, composed of 42 wt% of maize silage, 5 wt% of cow manure, and 53 wt% of cow sewage. Table 1 lists the main operational characteristics of the anaerobic digestion plant. SS-DIG was recovered from the solid-to-liquid separator (helical screw press). Once collected, a quantity of SS-DIG sample (referred to as “dry-SS-DIG”) was dried at 37 °C for 48 h. Another sample (referred to as “wet-SS-DIG”) was stored in gas-tight containers at 3 °C before being used. The initial solid digestate was composed of 22.4 (± 1.1) g TS/100 g of raw material and of 89.2 (± 2.3) g VS/100 g TS. 

Two grams (in equivalent of dry matter) of both dry SS-DIG and wet SS-DIG samples were milled using a vibro-ball mill “VBM” (MM400, Retsch, Düsseldorf, Germany) at a frequency of 20 s^−1^, at ambient temperature, for 5 or 30 min. For each condition, the experiment was repeated ten times to overcome heterogeneity of the SS-DIG samples. This apparatus consists of two 20-mL milling cups containing two stainless steel balls (2 cm diameter) each. 

### 2.2. Enzymatic Hydrolysis

Enzymatic hydrolysis of untreated and milled dry and wet-SS-DIG samples was performed using a mixture of enzymes (cellulase and endo-1,4-xylanase from *Trichoderma longibrachiatum*, Sigma-Aldrich^®^, Saint Louis, Missouri, United States) at 30 U/g TS, each. Enzymatic hydrolysis was carried out at a solids concentration of 50 g TS/L in 50 mM sodium acetate buffer (pH 5.0), 0.5 g TS L^−1^ chloramphenicol (Sigma–Aldrich^®^, Saint Louis, Missouri, United States) at 37 °C for 72 h, with stirring [12]. Each test was performed in triplicate. Liquid samples were withdrawn at 72 h and, after centrifugation, the supernatants were analyzed to determine the concentrations of monomeric sugars (i.e., glucose, xylose, and arabinose) using an HPLC (Waters Corporation, Milford, Connecticut, USA) equipped with a BioRad HPX-87H column at 40 °C, a refractive index detector at 40 °C, and 0.005 M H_2_SO_4_ as the solvent at 0.3 mL/min [12]. These analyses were performed in duplicate.

The cellulose and hemicelluloses hydrolysis yields were defined as follows:

(1) $$\mathrm{Cellulose}\;\mathrm{yield}\%=\frac{\left[\mathrm{Glucose}\right]}{1.111f}\times \;100\%,$$

(2) $$\mathrm{Hemicelluloses}\;\mathrm{yield}\%=\frac{\left[\mathrm{Xylose}+\mathrm{Arabinose}\right]}{1.136f}\;\times \;100\%,$$

where [Glucose] is the glucose concentration (g/100 g TS); [Xylose + Arabinose] is the xylose and arabinose concentration (g/100 g TS); and f is the cellulose or hemicellulose fraction in the dry biomass (g/100 g TS). 

### 2.3. Bioethanol Fermentation

The ethanol yields of raw and pretreated SS-DIG were evaluated and compared by simultaneous saccharification and fermentation experiments (SSF). The SSF tests were performed in duplicate on unsterilized samples, using 7 mL flasks (a working volume of 5 mL) sealed with rubber septa and equipped with an air vent system, comprising a sterilized needle and filter, in order to evacuate the CO_2_ produced during fermentation. A lyophilized yeast *Saccharomyces cerevisiae* strain was used as the inoculum. For this, lyophilized cells were washed and suspended in sterilized distilled water at a final concentration of 30 g TS L^−1^ [12]. Each flask contained: 0.25 g TS of sample (at 50 g TS L^−1^); cellulose and endo-1,4-xylanase enzymes (Sigma–Aldrich^®^, Saint Louis, Missouri, United States) at 20 U g^−1^ TS each; 0.25 mL of yeast (30 g TS L^−1^); and 0.5 mL of nutrient solution containing 50 g TS L^−1^ yeast extract (Yeast Extract, Technical, BactoTM, Becton, Dickinson and Company, Rutherford, NJ, USA), 4 g TS L^−1^ urea (Sigma–Aldrich^®^, Saint Louis, Missouri, United States), 0.5 g TS L^−1^ chloramphenicol (Sigma–Aldrich^®^ , Saint Louis, Missouri, United States), and 50 mM acetate buffer (pH 5.0). The flasks were incubated at 37 °C for 72 h with stirring at 500 rpm [12].

Samples (200 µL each) were withdrawn at 0, 2, 6, 24, 48, and 72 h and the cell-free supernatants were analyzed to determine the ethanol, cellobiose, glucose, xylose, and arabinose concentrations using an HPLC system (Waters Corporation, Milford, Connecticut, USA) equipped with a BioRad HPX-87H column at 40 °C, a refractive index detector at 40 °C, and 0.005 M H_2_SO_4_ as the solvent at 0.3 mL min^−1^. These tests were performed in duplicate.

Ethanol yields (g ethanol kg^−1^ TS) were calculated according to:

Ethanol yield (t) = [Ct ethanol / C solid] × 1000,(3)

where Ct ethanol (g ethanol L^−1^) is the concentration of ethanol produced during SSF, at time t; C solid (g TS L^−1^) is the total solids concentration in the flask.

### 2.4. Biochemical Methane Potential

Treated and untreated SS-DIG were digested in batch mesophilic anaerobic flasks. The volume of each flask was 1 L, with a working volume of 600 mL, the remaining 400 mL volume serving as headspace. SS-DIG and SS-DIG pretreated were introduced into the flasks with the inoculum ata substrate to inoculum ratio of 0.5 g VS/g VS. The inoculum was an inoculum acclimated in our laboratory with a feed composed of wastewater sludge and grasses. The inoculum contained 34 g/L total solids (TS) and 23 g/L volatile solids (VS). Once the flasks were prepared, degasification with nitrogen was carried out to obtain anaerobic conditions and the bottles were closed. Triplicate bottles were incubated at 35 °C. Assays with only inoculum were performed also in triplicate as blank control in the same condition. Biogas volume was monitored by pressure measurement using a manometer (Digitron 2023P, Digital Instrumentation Ltd., Corby, UK). Biogas composition was determined using a gas chromatograph (Varian GC-CP4900, Agilent, Ratingen, Germany) equipped with two columns: the first (Molsieve 5A PLOT, Agilent, Ratingen, Germany) was used at 110 °C to separate O_2_, N_2_ and CH_4_, the second (HayeSep A, Agilent, Ratingen, Germany) was used at 70 °C to separate CO_2_ from other gases. The injector temperature was 110 °C and the detector was 55 °C. The detection of gaseous compounds was done using a thermal conductivity detector. The calibration was carried out with two standard gasses composed of 35% CO_2_, 5% O_2_, 20% N_2_ and 40% CH_4_ for the first one and 9.5% CO_2_, 0.5% O_2_, 80% N_2_ and 10% CH_4_ for the second one (special gas Air Liquide^®^, Paris, France). All the results were expressed as biogas volume produced at normal conditions (in NL at 0 °C, 1013 hPa).

### 2.5. Analytical Determinations

Total Solids (TS), Volatile Solids (VS), and the ash content were analyzed according to a protocol used in previous studies [24]. The N content was determined with an elemental analyzer (VarioMacroCube, Elementar, Frankfurt, Germany). Protein levels were estimated by multiplying the N content by 6.25. Structural-carbohydrates from cellulose and hemicelluloses were measured using a strong acid hydrolysis method as described elsewhere [4]. All of the monosaccharides (i.e., glucose, xylose, and arabinose) were analyzed using an HPLC system (12620 Infinity II, Agilent, Ratingen, Germany) equipped with a column at 40 °C (Hi-Plex H, Agilent, Ratingen, Germany), a refractive index detector at 40 °C, and 0.005 M H_2_SO_4_ as the solvent at 0.3 mL/min. The system was calibrated with glucose, xylose, and arabinose standards (Sigma-Aldrich^®^, Saint Louis, Missouri, United States). The particle size distribution of untreated SS-DIG was determined using a vibratory sieving apparatus (Analytical Sieve Shaker AS 200, Retsch^®^, Düsseldorf, Germany) equipped with seven sieves of different sizes (2.5; 1; 0.71; 0.63; 0.45; 0.32 and 0.13 mm). The particle sizes of milled SS-DIG samples were analyzed using a laser granulometry (Mastersizer 2000, Malvern Instrument, Worcestershire, UK). Cellulose crystallinity (CrI) was determined using X-ray diffractometer (D8 Advance, Bruker, Aubrey, Texas, USA) as described elsewhere [26]. All of the analytical determinations were performed in duplicate.

### 2.6. Energy Balances of the Scenarios

Energy balances were computed by considering the additional electric and heat energy requirements for both scenarios: vibro-ball milling of dry SS-DIG and vibro-ball milling of wet SS-DIG. 

The thermal energy requirement (kWh_th_ kg^−1^ TS) for drying SS-DIG samples (E_th___drying_) before the vibro-ball milling process was calculated using Equations (4)–(8):

E_th___drying_ = E_Heat_ + E_Evaporation_, (4)

where E_Heat_ (kWh t^−1^ TS) is the energy requirement to increase the temperature of water and digestate from 25 °C to 100 °C, E_Evaporation_ (kWh_th_ day^−1^) is the energy of evaporation of water at 105 °C. E_Heat_ and E_Evaporation_ were calculated according to the equations below:

E_Heat_ = m × Cp × [T_Final_-T_Initial_]/3600, (5)

where m is the mass of water and digestate (equivalent to 1 kg of TS), Cp is the water specific heat (4.18 kJ kg^−1^ °C^−1^); T_Initial_ (°C) is the initial temperature of the substrate suspension, assumed to be 25 °C; T_Final_ (°C) is the final temperature of 105 °C.

E _Evaporation_ = [m_water_ × Lv]/3600, (6)

where Lv = 2380 kJ kg^−1^ and m is the mass of water in the digestate sample (equivalent to 1 kg of TS).

The electric energy requirement (E_el___milling_, kWh kg^−1^ TS) to mill SS-DIG was evaluated by using a watt-meter. The active power (watts), active electric energy (watts), frequency (hertz), and time (min) were logged into a PC card at 1-s intervals. The energy requirement was calculated according to Equation (7) below:

(7) $$\mathrm{Eel}\_\mathrm{milling}\;=\;\int_{0}^{t}(\mathrm{Pt})\mathrm{dt}\;/\;m,\;$$

where Pt is the power consumed in watts at time t and m is the mass of biomass to be ground in kg. Both Pt were measured in triplicate for each sample.

The energy efficiency (η, kg C6 _monomeric sugar_/kWh) was calculated using Equation (8):

(8) η = C6 Monomeric sugar yield / E,

where the monomeric sugar yield (kg/kg TS) is the amount of glucose produced during enzymatic hydrolysis of feedstock or digestate and E (kWh kg^−1^ TS) corresponding to the electrical and thermal energy requirements (Eel_milling and/or E_th___drying_). The two energy efficiency coefficients investigated were “ɳ_el_”, which is the ratio of soluble sugars (C6) and the electrical requirement, and “ɳ_tot_”, which is the ratio of soluble sugars (C6) and the overall energy requirement including both thermal and electrical requirements.

## 3. Results and Discussion

### 3.1. Physicochemical Characteristics of Untreated and Pretreated Digestate

The chemical compositions of untreated and VBM solid digestates, after mechanical screw separation, are listed in Table 2. There was a high volatile solids (VS) content of 89.2 g/100 g TS, mainly due to residual proteins and fibers (i.e., cellulose hemicelluloses, and lignin) that were not degraded during the AD process. The ash content was in accordance with the values obtained in our previous study [4], but lower than those of Ref. [27] who reported ash contents that varied from 23 to 38 g/100 g TS for four digestates derived from agricultural mesophilic AD plants that mainly treated manures, slurries, and silages [27]. The protein content (approximately 10.5 g/100 g TS), derived mainly by co-digestion of maize silage with cow manure and sewage, was in accordance with the values obtained by [4]. Similarly, Ref. [27] reported protein contents that varied from 10.5–12 g/100 g TS for various digestates from agricultural biogas plants [27]. In parallel, there was a large amount of residual carbohydrate polymers (cellulose, hemicelluloses), because during AD a large part of the biodegradable matter is not degraded, mainly due to physicochemical barriers that limit processing by bacteria [8,28]. In terms of the carbohydrate composition, there was a slightly higher cellulose content than hemicelluloses, as has also been observed by other authors who demonstrated that the anaerobic digestion process consumes hemicelluloses at a faster rate than cellulose [12]. Finally, there was a large amount of Klason lignin after the AD process (27 g/100 g TS). Such polymers are not degraded during the AD process and consequently accumulate in solid digestate [8,29]. 

**Figure 1:**
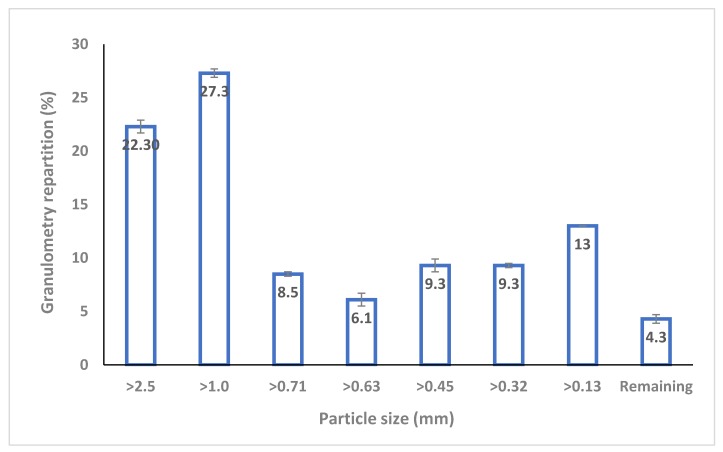
Granulometry repartition (in % of dry biomass) of the raw solid digestate.

In terms of the chemical composition (cellulose, hemicelluloses, lignin, and protein), no significant changes were observed before and after VBM of both dry and wet solid separated digestate (SS-DIG), as shown in Table 2. By contrast, VBM had a significant effect on the physical properties (i.e., particle size, CrI). First of all, VBM led to a significant decrease in the particle size of both dry and wet SS-DIG compared to untreated samples (Table 2 and Figure 1). However, there was a much more pronounced decrease in particle size with dry milling than with wet milling, thus confirming that the moisture content affects the decrease in particle size, as noted previously by [21]. Similar results were also noted by comparing dry and wet planetary ball milling as the pretreatment of *Pennisetum hybrid* [30]. Indeed, Ref. [30] found that dry milling had a greater impact on dry than wet biomass [D_50_ (µm)] of 131 µm and 243 µm were observed for dry and wet biomass, respectively, after 6 hours of milling) [30]. The results reveal that VBM pretreatment led to a reduction in the CrI index for dry SS-DIG, with a decrease in the CrI that varied from 27% to 75% after 5 min and 30 min of vibro-ball milling, respectively. Ref. [31] also observed a significant decrease in the CrI index after ball milling (30–120 min) of both sugarcane bagasse and straw [31]. Similarly, Ref. [23]reported significant decreases in the CrI of 55% and 96% compared to untreated sugarcane bagasse after 1 h and 3 h of VBM, respectively [23]. During the ball milling process, the shearing and compressive forces lead to a decrease in crystallinity [22,32,33]. By contrast, VBM had little or no impact on the CrI of wet SS-DIG. The lower degree of crystallinity of dry-milled biomass compared to wet may be due to the higher mechanical force from the dry milling process causing considerable change in the crystalline structure [32,34].

### 3.2. Enzymatic Hydrolysis

**Figure 2:**
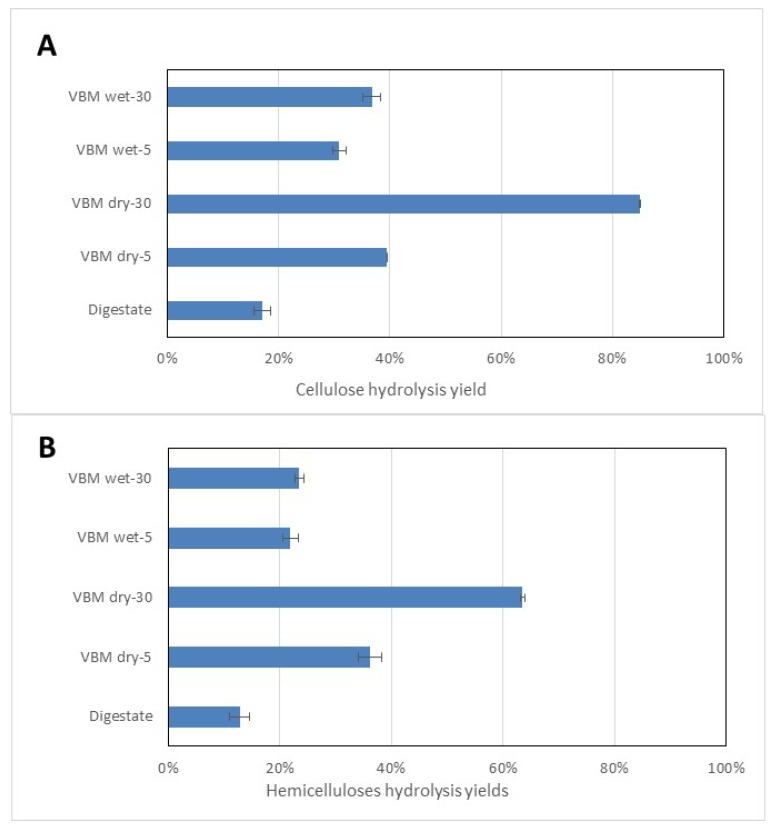
Enzymatic hydrolysis performances in terms of cellulose hydrolysis yield (**A**) and the hemicelluloses hydrolysis yields (**B**) for raw SS-DIG and vibro-ball milling (VBM)-pretreated samples in wet and dry modes.

The results show that VBM enhanced the enzymatic hydrolysis of the cellulose and the hemicelluloses fractions in both the dry and the wet mode (Figure 2). Raw digestate led to poor hydrolysis yields of cell carbohydrate polymers, with yields of 17% and 13% for cellulose and hemicelluloses, respectively. Similarly, a previous study reported that AD fibers were poor substrates for saccharification, with a sugar yield of only 12% of the original value on a dry basis [35]. In the case of VBM of dry SS-DIG, an increase in the duration of the pretreatment led to a higher level of enzymatic hydrolysis for both cellulose and hemicelluloses (Figure 2). Indeed, the yield of cellulose hydrolysis increased from 40% to 85% for 5 and 30 min of VBM, respectively. Similarly, the yield of hemicelluloses hydrolysis increased from 36% to 63% for 5 and 30 min of VBM, respectively. This improvement can be correlated to the decrease in both the crystallinity and the particle size when the duration of the ball milling of dry SS-DIG increased. This observation is supported by a number of authors who have demonstrated that during enzymatic hydrolysis of cellulose the readily accessible regions (i.e., the amorphous regions) are hydrolyzed more efficiently than the crystalline regions [36,37]. Furthermore, the surface area and the number of reactive sites increased due to reduction of the size of the substrate, thereby facilitating the adsorption of enzyme and hence the initial rate of hydrolysis [36]. This increase in enzymatic hydrolysis after mechanical processing has been reported previously in several investigations [23,31,38]. For instance, Ref. [23] reported a cellulose hydrolysis yield of 95% after 3 hours of VBM of sugarcane bagasse, whereas Ref. [39] reported a cellulose hydrolysis yield of 56% after 30 min of VBM of corn stover. The effect of VBM on wet digestate was less pronounced with a similar duration. Indeed, at 30 min of VBM, cellulose hydrolysis yields of 37% and 85% and hemicelluloses hydrolysis yields of 23% and 63% were obtained for wet and dry solid digestates, respectively. Such results can be explained by the lower impact of VBM on the particle size and the cellulose crystallinity of wet digestate, as indicated previously in Table 2. 

### 3.3. Energy Balance and Energy Efficiency Considerations

The total energy requirement for the drying and milling process of SS-DIG (Eel_milling and/or E_th___drying_) is presented in Table 3. A thermal energy requirement “E_th___drying_” of 3 kWh_th_ kg^−1^ TS was necessary to dry the solid digestate for further mechanical fractionation in dry mode. As demonstrated previously, if the biogas is converted by a CHP system, the excess heat produced during AD can meet the drying needs for the solid digestate [4,12]. In parallel, the electrical energy requirements (Eel_milling) for the VBM process were 9.9 and 54.4 kWh_el_ kg^−1^ TS for a duration of 5 min and 30 min, respectively. Similar values have been previously reported on various mechanical pretreatments even if the technologies used (e.g., ball milling, vibro-ball milling, hammer milling, and knife milling) and the substrates’ origin can influence the results [21,22]. For instance, Ref. [25] have reported energy requirements for dry ball milling of rice straw and consumption of 2.5 kWh_el_ kg^−1^ TS and 30 kWh_el_ kg^−1^ TS were observed for respectively 5 min and 30 min of pretreatment [25]. Some other mechanical systems (hammer mill, knife mill) have reported lower energy consumption on lignocellulosic biomasses but the final size particle of the substrates was higher than reported in our study [22,40]. In addition to enzymatic hydrolysis, in order to compare the various VBM modalities, the energy efficiency coefficients were calculated, as shown in Table 3 [41]. In general, the highest ɳ corresponds to the most effective pretreatment [39,41]. Both total sugar recovery and pretreatment energy efficiency should be used in evaluating and comparing the performance of pretreatment processes [41,42]. Interestingly, the best ɳ_el_ for the dry modality was obtained at VBM for 5 min, with a value of 0.102 kg sugars kWh_el_^−1^. Considering the thermal energy requirement as imput needed for SS-DIG drying, the energy efficiency ɳ_tot_ was reduced from 0.102 to 0.078 kg sugars kWh^−1^. Aside from a higher level of enzymatic hydrolysis of dry solid digestate at VBM for 30 min, the ɳ_el_ was worse due to the higher energy requirement for VBM. In regard to wet SS-DD, ɳ_el_ of 0.076 kg sugars kWh_el_^−1^ and 0.015 kg sugars kWh_el_^−1^ were noted for 5 min and 30 min of VBM, respectively. Such values are in agreement with previous one that reported ɳ_el_ from 0.011 to 0.078 kg sugars kWh_el_^−1^ after ball milling of rice straw for 60 and 5 min respectively [21,25]. 

In terms of industrial scale-up, the choice of the best scenario will be directly dependent on the on-site valorization of the biogas of the agricultural biogas plant. If the biogas is valorized through a CHP system, dry modalities of VBM can be pertinent for digestate valorization, as part of the heat excess from the cogeneration unit can be used to dry the digestate. In the case where biogas is upgraded into biomethane for gas injection, VBM to improve bioethanol or methane recovery from solid digestate has less merit. When optimizing VBM conditions, it is important to find a good compromise between the enzymatic hydrolysis performances and energy aspects represented by the coefficient of efficiency. As there was little difference between the ɳ_tot_ of dry SS-DIG at 5 min and 30 min, the 30 min conditions were hence selected for bioethanol production. 

### 3.4. Bioethanol Fermentation and Methane Potential

**Figure 3:**
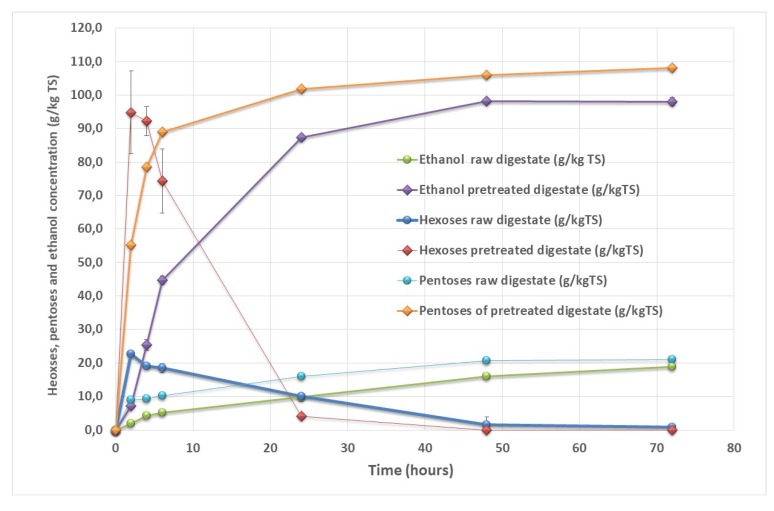
Hexose consumption and ethanol production for raw solid separated digestate and pretreated solid separated digestate with VBM for 30 min.

Bioethanol production was tested on the dry SS-DIG at the 30 min VBM conditions that led to the best level of cellulose hydrolysis (Figure 2 and Table 3). Bioethanol on raw digestate led to a low ethanol yield of 19 g_eth_ kg^−1^ TS corresponding to only 17% of the theoretical value, as shown in Figure 3. VBM of dry SS-DIG for 30 min led to an enhancement of the ethanol yield, with 98 g_eth_ kg^−1^ TS, corresponding to 90% of the theoretical value. Lower yields were reported by Ref. [18], with an ethanol yield of 37 g_eth_ kg^−1^ TS with dry SS-DIG that was mechanically pretreated (centrifugal milling). Other studies have demonstrated the benefit of pretreated SS-DIG to improve bioethanol production [14,15]. For instance, thermo-alkaline pretreatment has led to bioethanol recoveries from solid digestate of 75% to 80% compared to the theoretical value [13,14]. In future studies, it would be interesting to test other strains or bacteria capable of producing bioethanol from C_5_ sugar sources to improve bioethanol recovery from SS-DIG hydrolysate, as C_5_ sugars were not consumed in the present assay, as shown in Figure 3.

**Figure 4:**
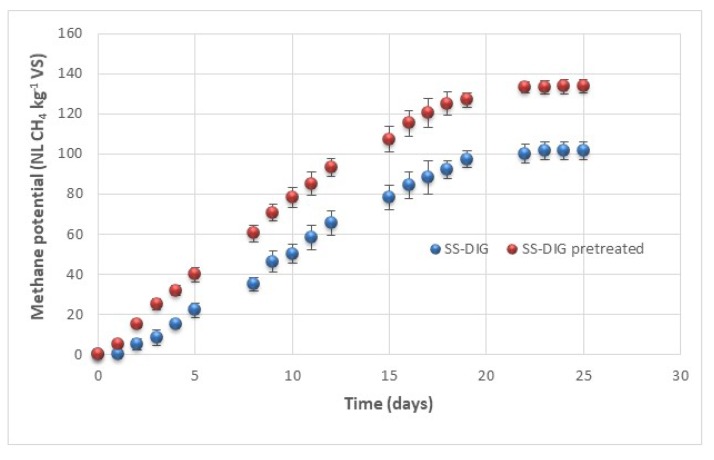
Methane potential (NL CH_4_ kg^−1^ VS) of raw SS-DIG and pretreated SS-DIG by VBM at 30 min.

In parallel, the impact of VBM pretreatment (for 30 min) have also been investigated on the methane potential of the SS-DIG in batch mesophilic conditions tests. Results are presented in Figure 4. SS-DIG exhibited a methane potential of 101.5 (± 4.3) NL CH_4_ kg^−1^ VS which is in agreement with previous studies that investigated the methane potential of solid separated digestate [7,18]. For instance, Ref. [18] have reported a methane potential of 90 (± 1.2) NL CH_4_ kg^−1^ VS for solid separated digestate from an agricultural biogas plant located in Italy. Similarly, Ref. [27] have reported methane potentials varying from 71.4 (± 5.3) NL CH_4_ kg^−1^ VS to 156.9 (± 7.4) NL CH_4_ kg^−1^ VS for three solid separated digestates from agricultural biogas plant. Results were also found in accordance with those of Ref. [7] who investigated the residual methane potential of whole digestate from 21 full-scale digesters and they reported methane yields varying from 24 to 126 NL CH_4_ kg^−1^ VS. The application of VBM pretreatment on the dry solid separated digestate has permitted significant improvement of the methane potential. Indeed, a methane potential of 133.6 (± 3.3) NL CH_4_ kg^−1^ VS was observed corresponding to an improvement of the methane potential of 31% compared to untreated SS-DIG. Such results are in agreement with previous ones that have highlighted the benefits of applying a post-treatment to improve methane recovery from the SS-DIG [18,27]. For instance, Ref. [27] have reported that thermal pretreatment (120 °C, 30 min) lead to an improvement of the methane potential from 71.4 (± 5.3) NL CH_4_ kg^−1^ VS to 153.7 (± 20.6) NL CH_4_ kg^−1^ VS from the solid separated digestate of an agricultural biogas plant fed with swine slurry, grass silage and maize silage. Similarly, Ref. [43] reported an improvement of the methane potential of a solid separated digestate from a full-scale biogas plant from 21 (± 0.0) NL CH_4_ kg^−1^ VS to 57.7 (± 4.5) NL CH_4_ kg^−1^ VS after 10 min of ball milling pretreatment. 

According the results obtained, it is obvious that the vibro-ball milling is a promising technology for improving the biodegradability of the SS-DIG and improving the recovery of bioethanol and methane. In future work, such results have to be confirmed at pilot-scale as the energy requirement of the milling step is often overestimated at laboratory-scale and can be used only as rough estimation for the optimization of the operational parameters. Currently, the pretreatment of the SS-DIG for further recirculation into the AD process seems to be simpler and more interesting from an energetic point of view (1.22 kWh kg^−1^ TS for AD process compared to 0.72 kWh kg^−1^ TS for bioethanol production). Nonetheless, AD/bioethanol answers better to the prerequisites of a biorefinery, by diversifying biofuels production (biogas, bioethanol).

## 4. Conclusions

VBM appears to be a promising technology to improve sugar recovery after enzymatic hydrolysis from dry solid anaerobic digestate. VBM of dry anaerobic digestate led to a significant decrease in the particle size and crystallinity. The best results in terms of cellulose and hemicelluloses hydrolysis were noted with VBM for 30 min, with hydrolysis yields of 64% and 85% for hemicelluloses and cellulose, respectively. Bioethanol fermentation (SSF) under this condition led to an ethanol yield of 98 g_eth_ kg^−1^ TS (corresponding to 90% of the theoretical value) compared to 19 g_eth_ kg^−1^ TS for raw solid digestate. In parallel, VBM for 30 min led to an improvement of the methane potential of 31% compared to untreated SS-DIG.

## Figures and Tables

**Table 1:** The main operational characteristics and performances of the agricultural biogas plant unit.

Anaerobic Digester Parameters	
Number of reactors	2 digesters, 1 post-fermenter, 1 storage tank
Reactors Volume (m^3^)	Digesters: 2 × 2400 Post-fermenter: 2700 Storage tank: 2700
Feeding (t FM day^−1^) ^a^	95
HRT (d) ^a^	80
pH ^a^	7.9
Temperature (°C) ^a^	40
Biogas	
Biogas (Nm^3^ day^−1^)	12,000
Methane (%)	53
Total Energy (kWh day^−1^) ^b^	63,600

^a^ Referred to digesters and post-fermenter; ^b^ 10 kWh Nm^−3^ methane.

**Table 2:** Physicochemical characteristics of untreated and pretreated digestate by vibro-ball milling of dry and wet solid separated digestate (SS-DIG).

Parameters	SS-DIG	VBM Dry—5 min	VBM Dry—30 min	VBM Wet—5 min	VBM Wet—30 min
Volatile Solids (g/100 g TS)	89.2 ± 2.3	91.3 ± 0.4	91.7 ± 0.5	91.0 ± 0.2	90.0 ± 0.2
Proteins (g/100 g TS)	nd	10.5 ± (0.1)	9.9 ± (0.3)	9.7 ± (0.6)	10.8 ± (0.0)
Cellulose (g/100 g TS)	nd	22.9 ± (3.1)	20.2± (0.4)	22.2 ± (1.5)	21.1 ± (1.5)
Hemicelluloses (g/100 g TS)	nd	16.6 ± (1.9)	16.5 ± (0.2)	16.9 ± (1.5)	17.9 ± (2.2)
Lignin (g/100 g TS)	nd	26.9 ± (0.3)	28.2± (0.8)	26.3 ± (0.3)	29 ± (1.3)
Ash (g/100 g TS)	10.1 ± 1.9	8.2 ± (0.4)	8.1 ± (0.9)	10.1 ± (1.9)	9.9 ± (0.2)
Particle size (D50, µm)	-	35 (± 7)	48 (± 2)	202 (± 28)	162 (± 34)
Cri (%)	44	32	11	46	41

nd = not determined.

**Table 3:** Electrical and thermal energy requirements and energy efficiencies of the various milling modes applied to dry and wet SS-DIG.

	Electrical Consumption (kWh_el_ kg^−1^ TS)	Thermal Energy (kWh_th_ kg^−1^ TS)	C6 Sugars (g kg TS^−1^)	Efficiency ɳ_el_ (kg Sugars kWh_el_^−1^)	Efficiency ɳ_tot_ (kg Sugars kWh^−1^)
SS-DIG	-	-	44		
VBM dry—5 min	9.9	3.0	101	0.102	0.078
VBM dry—30 min	54.4	3.0	191	0.035	0.033
VBM wet—5 min	9.9	-	76	0.076	0.076
VBM wet—30 min	54.4	-	86	0.015	0.015

## Abbreviations

AD	Anaerobic digestion
CHP	Combined Heat and Power
Cp	Specific heat of water
E_DY_	Energy requirement for drying
E_Heat_	Energy requirement for heating
E_Evaporation_	Energy requirement for evaporation
Lv	Latent heat of vaporization
HRT	Hydraulic Retention Time
SS-DIG	Solid Separated Digestate
TS	Total Solids
VS	Volatile Solids
wt.	Weight
